# Dr. Carson, on the Use of Calomel

**Published:** 1800-11

**Authors:** William Carson

**Affiliations:** Practitioner in Midwifery. Birmingham


### *To the Editors of the Medical and Phyfical Journal.*

GENTLEMEN,

FOR feveral years I have been diffatisfied with the general and indifcriminate ufe of Calomel in the difeafes of children; I am not more certain of any one fact that pertains to medicine, than that I have feen many children who have fallen a facrifice to the improper application of this medicine. Calomel when mixed with fugar, forms a medicine agreeable to the palate of the child; its exhibition is eafy to the mother or nurfe, and it may with fafety be given as a purge, when a purge is indicated. When given as a purge, its action is confined to the firft paffages, but when the dofe is frequently repeated, either for the purpofe of obviating habitual coftivenefs, or with any other intention, it is abforbed by the lymphatics, and enters the fyftem, by the action of which it is decompofed. The oxygene difengaged from the mercury, forms a powerful ftimulus to the fyftem, increafing the action of all its irritable fibres: the the action of the system thus increased, produces an increased decomposition of the oxygene gas absorbed by the lungs; this decomposition is attended with an increase of animal heat, which, in its turn, acts as an additional stimulus, and that state of the system is produced, which is called the *mercurial fever*; this fever, in adults, soon runs into indirect debility. Although mercury does not appear to have so powerful an action on the salivary glands of children as it has on adults, yet, I apprehend its general effects upon the system are greater.

The irritability of an infant is to that of a person in middle age as 2 to 1: the pulse of the latter is from 60 to 80 strokes in a minute; the former from 120 to 140. The increase of excitation produced on the fibres of an infant by an increase of stimulus, will be double the excitation produced on the fibres of an adult from the same increase of stimulus, and the increase of indirect debility will be in the same proportion.

The injurious consequences likely to flow to children from the high degree of excitation, and extreme succeeding debility produced by a mercurial course, I wish to impress upon your readers. Mercury has been erroneously held forth as a specific in hydrocephalus, and is often given as a preventive of that fatal malady: Hydrocephalus appears to be the result of debility succeeding too high an action of the vessels in the brain. If so, can any medicine more powerfully produce hydrocephalus than mercurial calces? I am, GENTLEMEN,

Your very humble servant,

WILLIAM CARSON, M. D.*
Practitioner in Midwifery.

*Birmingham,*

*July* 21, 1800.

### *To the Editors of the Medical and Physical Journal.*

GENTLEMEN,

SHOULD you think the following mode of treating the residuum of vinegar, in the process Acetum Distillatum of the Edinb. College, merits a place in the Journal, please to insert it.

*Edinburgh, Aug.* 13, 1800. B. B. SIMONS, M. D.

THE College orders "eight pounds of vinegar to be distilled in glass vessels with a gentle heat; let the two first pounds that

* Dr. Carson will soon present the public with a work on the *Treatment of Parturient Females, chiefly with a view to prevent abortion.*

